# Editorial: Machine learning and immersive technologies for user-centered digital healthcare innovation

**DOI:** 10.3389/fdata.2025.1567941

**Published:** 2025-02-14

**Authors:** Federico Colecchia, Daniele Giunchi, Rui Qin, Eleonora Ceccaldi, Fang Wang

**Affiliations:** ^1^Brunel Design School, Brunel University of London, Uxbridge, United Kingdom; ^2^Department of Computer Science, University College London, London, United Kingdom; ^3^School of Computing and Mathematical Sciences, University of Leicester, Leicester, East Midlands, United Kingdom; ^4^CasaPaganini - InfoMus, DIBRIS, University of Genoa, Genoa, Liguria, Italy; ^5^Department of Computer Science, Brunel University of London, Uxbridge, United Kingdom

**Keywords:** digital innovation for health and wellbeing, interdisciplinary collaboration, artificial intelligence, immersive technologies, machine learning, virtual reality, augmented reality, mixed reality

## User-centered design for digital healthcare innovation

Modern digital technologies such as machine learning and immersive technologies, including virtual reality and augmented reality, hold potential for enabling disruptive innovations to promote individuals' health and wellbeing. However, the adoption of such technologies, including the use of data-driven tools to support healthcare professionals' decision-making and applications relying on consumer electronic devices for the benefit of individuals, is often hindered by issues that do not necessarily arise from technological limitations but rather are user-centered in nature. Whether new technologies become successfully embedded within individuals' daily routines and professionals' workflows often depends on the way in which ethical issues directly impacting on user trust have been addressed at the design concept generation, development, and deployment stages.

There is increasing recognition of a need to facilitate further convergence between the development of emerging technologies for promoting individuals' health and wellbeing and user-centered design research, with a view to achieving positive impact on individuals, care professionals, and healthcare systems. In addressing current development trends relating to user-centered digital innovation for health and wellbeing based on machine learning and immersive technologies, this Research Topic across Frontiers in Artificial Intelligence, Frontiers in Virtual Reality, and Frontiers in Big Data has attracted 13 contributions including original research articles, reviews, perspectives, as well as theoretical and methodological contributions, thereby providing a snapshot of recent and ongoing research and development.

## Machine learning and immersive technologies: a case study

Whereas the Research Topic title explicitly refers to healthcare innovation, the broader scope has turned this article collection into an opportunity for reflection on a range of topics of relevance to both health and wellbeing. Topics have included the use of technologies for enhancing the provision of medical education and training, for improving workforce wellbeing, and for augmenting art therapy programmes with a view to increasing therapeutic compliance. Methods employed include Agile data science techniques, “CRoss Industry Standard Process for Data Mining” (CRISP-SM), “Preferred Reporting Items for Systematic reviews and Meta-Analyses” (PRISMA), thematic literature review, mini review, primary research (specifically, collection of data from university nursing students and surgeons), “Simulation Effectiveness Tool – Modified” (SET-M), established data science techniques, and human factors engineering methods. Key research themes that have emerged from the Research Topic are discussed below, followed by a reflection on priorities for further research and development.

### Interdisciplinarity, ethics, and stakeholder engagement

The articles have highlighted a need for knowledge and expertise from across academic disciplines and professional practice to converge and underpin the development of digital innovations promoting individuals' health and wellbeing. Relevant disciplines and domains include computer science, user-centered design, human-computer interaction, engineering, human factors engineering, and the social sciences. Technology end user and stakeholder values, expectations, and requirements need to be addressed if new methods enabled by modern digital technologies are to be sustainably employed (Kolyshkina and Simoff; Goisauf and Cano Abadía; Khatun et al.; Buche et al.). Interestingly, the articles have generally suggested the importance of embedding end user and stakeholder perspectives within technology development workflows, although only a minority of the studies have explicitly articulated the need for extensive involvement of human-centered design researchers and practitioners for scaffolding and facilitating iterative development and evaluation (Khatun et al.). Unsurprisingly, the need to address ethical concerns appears intertwined with the recognized desirability of research objectives and methods to deliver deeper integration across discipline boundaries. This is illustrated by research advocating the adoption of intersectional social sciences perspectives within AI development for cancer diagnostics (Goisauf and Cano Abadía) and by studies focusing on the representativity of machine learning training data with a view to reducing health inequalities affecting specific ethnic groups in relation to the provision of diagnostic services (Khatun et al.).

### Production-ready systems

The design and development of production-ready AI-based systems designed for flexibility and maintainability over time have received significant attention in recent years, particularly in the information systems, human-computer interaction, and engineering design literature. This is illustrated by Research Topic articles focusing on the definition of architectural requirements for healthcare cost estimation systems relying on dedicated predictive numerical models (Jackson et al.), and by studies delivering prototype models to enable healthcare professionals to query different Electronic Medical Record systems using intuitive interfaces based on natural language (Marshan et al.). Such efforts have achieved a balance between adapting research pipelines for production environments and identifying optimized architectural specifications from an information systems perspective.

### Medical education and training

The emphasis in recent academic and professional discourse on opportunities afforded by immersive technologies, including virtual reality, augmented reality, and mixed reality, for achieving more efficient and inclusive delivery of medical educational and training programmes is reflected in this article collection. An interesting review study has focused on a comparison between technology-augmented methods and established approaches (Xu et al.). Reported benefits include enhanced student and trainee motivation, satisfaction, and learning outcomes, although the possible occurrence of undesired consequences of the use of immersive technologies, including cybersickness following prolonged exposure, has been noted. Interestingly, one study has focused on real-time detection of cybersickness with a view to reducing detrimental effects on user experience (Yalcin et al.). Proposed innovations based on mixed reality to streamline urology anatomy training and to facilitate pre-operative urology planning have attracted positive feedback from both university nursing students and surgeons, which encourages further research toward more extensive clinical validation (Sánchez-Margallo et al.). An interesting study has focused on an integration between generative AI and immersive technologies for designing augmented reality filters, with a view to improving medical students' perceptions of self-efficacy in recognizing selected disease manifestations (Stuart et al.).

### Individuals' wellbeing

The potential of modern digital technologies for improving individuals' wellbeing has been the subject of recent research, which is reflected in this article collection. The breadth of contributions received illustrates the potential of artificial intelligence and immersive technologies for improving individuals' wellbeing, particularly in clinical and workplace settings. A theoretical model has been presented, explaining the psychological benefits of virtual immersion for oncology patients with emphasis on distraction for alleviating anxiety and pain (Buche et al.). Opportunities have been identified for artistic expression within virtual reality environments to increase therapeutic compliance and to improve wellbeing outcomes for individuals in relation to psychotherapy and neurorehabilitation (Hadjipanayi et al.). A study has identified features of immersive technologies that hold potential for improving motor rehabilitation compliance and efficacy with stroke patients when used in combination with traditional approaches (Bui et al.). Such features include those enabling real-time movement tracking and the provision of reinforced feedback in line with established neurorehabilitation principles. A review of modern digital technologies for estimating individuals' wellbeing in workplace settings has generated useful recommendations on how real-time posture detection techniques can best be combined with the adoption of established human factors engineering best practices (Ataguba and Orji). This has enabled the identification of optimized algorithms to be employed in conjunction with physiological sensing methods toward the design of healthier workplaces.

## Promoting a design-driven user-centered research and development agenda

Overall, the articles published under this Research Topic have highlighted the importance of conducting interdisciplinary research when tackling challenges at the intersection of technology development with human-centered design and human factors engineering. Integrative capabilities across academic disciplines and research methodologies—with emphasis on modern design research and design professional practice—have been identified as an important enabler of challenge-driven research and responsible innovation. Such insights are relevant to the United Nations Sustainable Development Goal number 3 (“Good health and wellbeing”) and more broadly (Colecchia et al., 2024). A balanced distribution has been achieved in this collection of articles between applications of immersive technologies (Buche et al.; Xu et al.; Yalcin et al.; Sánchez-Margallo et al.; Stuart et al.; Hadjipanayi et al.; Bui et al.) and applications of machine learning and artificial intelligence (Kolyshkina and Simoff; Goisauf and Cano Abadía; Khatun et al.; Jackson et al.; Marshan et al.; Yalcin et al.). The authors speculate that future research is likely to reflect a convergence of immersive technologies and artificial intelligence in relation to the promotion of individuals' health and wellbeing. If that is the case, it is anticipated that the emphasis will be on human-centered design, participatory design, and methods addressing ethical issues of privacy, transparency, equitability, and fairness. One potential area of convergence relates to the development of personalized immersive experiences designed for inclusivity. It is expected that reliance on human-centered and participatory design methods will prove useful for scaffolding iterative design with significant involvement of technology end users and stakeholders. This is illustrated by the article discussing the use of artistic experiences within virtual reality environments to increase therapeutic compliance and to improve wellbeing outcomes (Hadjipanayi et al.). The articles have also highlighted several limitations with the technology state of the art, which calls for additional emphasis on interdisciplinary research, human-centered design, and inclusive design research moving forward. Such limitations include the following: digital access barriers and reduced digital literacy across user groups; the generally reduced availability of dedicated features for visually-impaired individuals—and for individuals with specific characteristics more broadly—compared with mainstream users; undesired effects from the use of head-mounted immersive displays—including cybersickness; the presence of different skill sets within interdisciplinary AI development teams, potentially reducing the benefits of Agile development. Moreover, although the articles published under this Research Topic have not focused on this aspect, future development will also need to address elements of clinical validation of digital technologies in the context of the relevant regulatory frameworks.

The authors argue that higher education institutions should take the lead in promoting long-term sustainable innovation in collaboration with research, healthcare, and commercial organizations. Non-academic organizations are sometimes better positioned for liaising with technology end users and practitioners, whose involvement in participatory design activities should be promoted wherever possible—ideally starting with the generation of early-stage design concepts. On the other hand, higher education institutions are ideally positioned to contribute expert knowledge and should lead on challenge-driven interdisciplinary research and on the promotion of postgraduate collaborative student projects. The emphasis should be on facilitating transfer of knowledge about modern human-centered design methods to non-academic organizations, with a view to creating favorable conditions for the achievement of broader and sustainable positive impact of research on individuals, society, and the economy.
